# Gender Differences in Drinking Practices in Middle Aged and Older Russians

**DOI:** 10.1093/alcalc/agq069

**Published:** 2010

**Authors:** Natalia Bobrova, Robert West, Darya Malyutina, Sofia Malyutina, Martin Bobak

**Affiliations:** 1 Department of Epidemiology and Public Health, University College London, 1-19 Torrington Place, London WC1E 6BT, UK; 2 Institute of Internal Medicine, Siberian Branch of the Russian Academy of Medical Sciences, Novosibirsk, Russia

## Abstract

**Aims:** The study investigated gender differences in drinking patterns and the reasons behind them among men and women in the Russian city of Novosibirsk. **Methods:** A mixed method, combining quantitative and qualitative data, was conducted based on the Health, Alcohol and Psychosocial factors In Eastern Europe cohort study. The quantitative study included 4268 men and 5094 women aged 45–69 years; of those, 20 men and 24 women completed an in-depth interview. **Results:** The quantitative data revealed a large gap in drinking patterns in general between genders. Women drank less often and much smaller quantities than that of men. For example, 19% of men, vs. 1% of women, were classified as problem drinkers (two or more positive answers on the CAGE questionnaire). These differences were not explained by socioeconomic factors. Qualitative data have shown that gender roles and a traditional culture around women's and men's drinking were the main reasons for the reported drinking behaviour, whereby women were consistently expected to drink much less than men in terms of preference for strong beverages, drinking frequency and quantity of alcohol consumed. **Conclusion:** The study confirmed that large differences exist between Russian men's and women's drinking; these differences may be largely explained by gender roles.

## INTRODUCTION

The mortality crisis in Russia that occurred just after the collapse of the Soviet Union in the early 1990s attracted the attention of many researchers and demographers (Leon *et al*., 1997; Notzon *et al*., 1998; Shkolnikov *et al*., 1995). Mortality was already increasing in the Soviet Union from the 1960s, especially among males, leading to a growing gap between the life expectancy of men and women (Treml, 1976). At present, the gender gap in life expectancy at birth in Russia is 12 years, one of the largest in the world. The causes of the gender gap in mortality are complex, almost certainly including tobacco (Bobak, 2003) and other lifestyle factors (United Nation Development Program (UNDP), 2005). However, there is increasing evidence that alcohol contributed to the high mortality rates in Russia (Leon *et al*., 2007, 2009; Pridemore, 2002; Pridemore and Chamlin, 2006; Zaridze *et al*., 2009), and it is likely that alcohol also contributed to the large gender gap in mortality.

This hypothesis is supported by observations that the differences in alcohol consumption between genders are much greater in Russia than in other countries. For example, in a Moscow-based study, women reported three times fewer drinking occasions than men, and only one percent of women drank to a high level of intoxication, compared with 19% of men (Simpura and Levin, 1997). In a prospective cohort study in the USA and Russia in 1972–1982, 27% of Russian women and 68% of Russian men reported having at least one drink during the last week, compared with 53% of American women and 72% of American men (Deev *et al*., 1998). A more recent study in Novosibirsk confirmed the large gender differences in drinking in Russia: 30% of Russian men, compared with only 1% of women, reported binge drinking at least once a month (Bobak *et al*., 2004). Gender differences of a similar magnitude were found in problem drinking, based on the alcohol-related problems, screening instrument, CAGE and negative social consequences of drinking (Bobak *et al*., 2004). This pattern of differences in drinking between men and women is consistent with other studies in Russia (Bobak *et al*., 1999; Malyutina *et al*., 2001; Pomerleau *et al*., 2008).

Although there is some evidence that women's drinking may be under-reported in population studies in Russia (Laatikainen *et al*., 2002), the extent of under-reporting is unlikely to explain the large differences in drinking behaviour between men and women found repeatedly in population-based studies in Russia. The aim of the present study was to describe drinking patterns of men and women randomly selected from the Novosibirsk population (using quantitative data) and to explore the reasons for such differences (using qualitative data). The qualitative study used semi-structured interviews to provide additional understanding of particular behaviours and to elucidate issues, which are difficult to pick up in epidemiological surveys (Heath, 1998; Strunin, 2001).

## METHODS

We conducted a mixed design study, combining quantitative survey and qualitative data, using the Novosibirsk part of the Health, Alcohol and Psychosocial factors In Eastern Europe (HAPIEE) study, which explores the trends in cardiovascular disease and mortality and investigates their determinants in urban populations.

### Quantitative survey

The HAPIEE project includes four cohorts of random samples of men and women aged 45–69 selected from population registers in Novosibirsk (Russia), Krakow (Poland), Kaunas (Lithuania) and six Czech towns. A detailed description of this cohort study including the survey and data collection procedures has been presented elsewhere (Peasey *et al*., 2006). The present study was based on the Novosibirsk cohort baseline survey, which included in total 4269 men and 5094 women. The survey was conducted between 2002 and 2005, and the participants were interviewed in a clinic by trained female nurses. The structured questionnaire covered eight major areas: social status, health, physical functioning, psychosocial factors, economic factors, retirement, quality of life, and community and social capital characteristics.

### Drinking measures

To describe alcohol consumption patterns including problem alcohol intake, we used the graduated frequency questionnaire (GF; Greenfield, 2000; Rehm, 1998). The questionnaire asked about the amount of alcohol in local units of beer, wine or spirits, and the number of times it was consumed in the past 12 months. The amount of alcohol ranged from about half a drink to 10 or more drinks. One drink was defined as 500 ml of beer, 200 ml of wine or 50 ml of spirits. Data from GF allowed the estimation of mean annual number of drinking occasions, annual alcohol intake, mean dose of alcohol per drinking session, and the frequency of drinking and binge drinking. We defined *'*binge drinking' as drinking five or more drinks per session at least once a month for men (which is equal to ∼100 g of pure ethanol) and three or more drinks per session at least once a month for women (equivalent of ∼60 g of ethanol). Similar cut-off points were previously used in many studies on different populations, including Russia (Bobak *et al*., 1999, 2004; Malyutina *et al*., 2001, 2002; National Institute on Alcohol Abuse and Alcoholism, 2004). The questionnaire also asked about typical weekly intakes of beer, wine and spirits; from which the total weekly alcohol intake (in grams of ethanol) was calculated.

### Negative consequences of drinking

We used the CAGE questionnaire (Ewing, 1984); people with two or more positive answers to each questionnaire were considered as problem drinkers. We also included eight questions on problems caused by alcohol in different areas of life in the past 12 months (Bobak *et al*., 2004); again, two or more positive answers were considered to indicate negative consequences of drinking.

### Covariates

Several other variables were used in the analyses. Education was coded into four categories (primary, vocational, secondary and university). Self-reported material deprivation was measured by three questions on difficulties buying enough food and clothes and paying bills; the answers (on a 4-point scale) were summed, and the score (ranging from 0 to 12) was categorized into four groups (0, 1–2, 3–5 and 6–12). Marital status was classified in four groups (married/cohabiting, single, divorced and widowed) and history of unemployment was recorded also in four groups (never, <3 months in total, 3–11 months and 12 or more months).

### Statistical analysis

First, we compared the crude available measures of drinking between men and women. Second, we used linear and logistic regression to assess the significance of differences in various drinking indices between genders in age-adjusted models and in models additionally adjusted for marital status, unemployment history, education and material deprivation.

### Qualitative study

We conducted 44 qualitative semi-structured in-depth interviews among 20 men and 24 women conveniently sampled from the HAPIEE cohort to produce ‘detailed descriptions’ of drinking patterns from the participants’ own perspectives. Potential participants were approached in the clinic prior to or after their examination for the main study during the second wave data collection. They were asked whether they would agree to participate in tape-recorded interview that would explore drinking patterns in Russia, and those who provided consent were recruited to the study. There were no refusals.

### Qualitative interviews

Interviews were conducted on several occasions between May 2006 and November 2007. Interviews lasted between 50 and 90 min and were tape recorded with participants’ consent. Interviews took place at the study clinic in a private room. All tape-recorded interviews were transcribed in Russian verbatim. The data were analysed using the framework approach and inductive and thematic techniques to allow frequently reported patterns to emerge from the raw data. (Richie and Spencer, 1994; Richie *et al*., 2003). Two independent researchers coded transcribed interviews line-by-line by identifying themes and categories. The coding frame was developed after a preliminary analysis of a subsample of transcripts, and then was applied to the whole data.

Male and female respondents were asked directly about their opinions of how men and women drink. For example: are there any differences, what kind of differences are there and what are the reasons for them? Both male and female respondents were asked to describe the drinking of their significant others (there were no couples interviewed in the study), relatives and peers of the opposite sex. Although it was not directly imposed on participants (as it was anticipated that data will flow from the conversations themselves and will not be suggested by the interviewer), traditional values, social contexts and social roles between genders were explored across interviews.

## RESULTS

### Survey data

#### Socio-demographic characteristics

In total, 4269 men and 5094 women were included in the study (Table 1). The mean age of both men and women was 58 years. There were differences between men and women in their marital status (88% of men vs. 60% of women were currently married), education (more men than women in vocational category), material deprivation (more women than men were in the most deprived category) and history of unemployment (slightly more women were in categories with longer history of unemployment).

**Table 1.** Socio-demographic characteristics and alcohol consumption and drinking frequency of men and women in the quantitative study

	Men (*n* = 4269)	Women (*n* = 5094)	*P*-value
Age, mean (SD)	57.8 (7.0)	57.6 (7.1)	0.071
Education (%)			
Primary	11.40	9.60	
Secondary	35.00	33.60	
Vocational	21.70	30.50	<0.001
University	31.90	26.30	
Marital status (%)			
Married/cohabiting	87.80	59.40	
Single	2.60	4.70	<0.001
Divorced	5.60	14.50	
Widowed	4.00	21.4	
Deprivation (%)			
1 (lowest)	37.40	20.00	
2	15.40	14.50	
3	20.80	26.50	<0.001
4 (highest)	26.40	39.00	
History of unemployment (%)			
Never	80.20	78.80	
Up to 3 months in total	6.00	3.70	<0.001
4–11 months in total	5.80	6.50	
12 months or more in total	8.00	11.00	
Drinking frequency in last year (%)			
Never	13.50	17.90	
Less than once a month	17.70	55.50	
1–3 times a month	24.10	18.90	<0.001
1–4 times a week	36.60	7.40	
5 or more times a week	5.10	0.40	
Annual alcohol intake (%)			
None	13.50	17.90	
<3 l of pure alcohol	44.90	79.40	
3–5.99 l of pure alcohol	14.00	1.60	<0.001
6–11.99 l of pure alcohol	16.70	0.90	
12 l or more of pure alcohol	11.00	0.10	
Problem drinking (CAGE score ≥2)	19.20	1.40	<0.001
Negative consequences of drinking	9.10	1.80	<0.001
Binge drinking^a^	29.90	6.10	<0.001

^a^Men: ≥100 g at least once a month; women: ≥60 g at least once a month.

### Drinking patterns

A crude comparison of different indicators of drinking in men and women is also shown in Table 1. A significantly larger proportion women (17.9%) than men (13.5%) did not drink alcohol in the last 12 months. Drinking frequency also differed between genders, with drinking at least once a week being reported by 52% of men and only 9.5% of women. In contrast, 68% of women and 20% of men reported drinking less than once a month. The most common frequency of drinking reported by men was drinking 1–2 times a week, compared with 6–11 times a year among women. The annual intake of alcohol was considerably higher in men than in women. There were also large differences in the prevalence of negative consequences of drinking, problem drinking and binge drinking between the genders.

Table 2 shows the differences in different drinking indices between men and women after adjusting for age and after further controlling for socioeconomic factors. All comparisons show much higher levels of drinking in men, and these differences are not explained by differences in socioeconomic circumstances of men and women. For example, the annual intake of alcohol was more than 5 l higher in men than in women, and this estimate was similar in Models 1 and 2.

**Table 2.** Odds ratios and differences in drinking indices between men and women in the quantitative study, in models adjusted for age (model 1) and for age, education, deprivation, marital status and history of unemployment (model 2)

	Men	Women	Men vs. women Model 1	Men vs. women Model 2
Binary variables	%	%	OR (95% CI)	OR (95% CI)
Drinking at all	86.6	82.1	1.4 (1.3–1.6)	1.3 (1.2–1.5)
Drinking at least once a week	44.7	7.8	10.0 (8.9–11.3)	9.5 (8.4–10.8)
Drinking at least 6L per year	27.7	1.1	36.8 (28.0–48.5)	38.6 (29.1–51.3)
Problem drinking (CAGE core ≥2)	19.2	1.4	16.9 (13.2–21.5)	18.4 (14.3–23.8)
Negative consequences of drinking	9.1	1.8	13.2 (9.5–18.4)	16.2 (11.4–23.0)
Binge drinking	29.9	6.1	6.9 (6.0–7.9)	7.5 (6.5–8.7)
Continuous variables, among drinkers	Mean (SD)	Mean (SD)	Diff. (95% CI)	Diff. (95% CI)
Annual intake (litres of ethanol)	5.8 (8.8)	0.6 (1.8)	5.3 (5.1–5.6)	5.5 (5.2–5.8)
Mean dose (grams of ethanol)	63.1 (43.8)	25.9 (15.9)	37.6 (36.2–39.0)	39.4 (37.8–40.9)
Weekly alcohol intake (grams of ethanol)	89.7 (154.8)	5.9 (23.5)	85.3 (80.6–90.1)	87.8 (82.7–93.0)

### Qualitative study

We interviewed 20 women and 24 men aged from 48 to 63, selected from the quantitative study described above. Most women in the sample reported drinking only on special occasions. Men reported a greater variety of patterns including drinking once a month, drinking weekly and drinking daily. We had several abstainers in the sample. Male abstainers were previously very heavy drinkers but because of poor health and family reasons had become abstainers. One woman was a life-time abstainer (never tried alcohol), and another one was ‘a light drinker’ [pochti ne pila] before and stopped drinking because of poor health.

The preferences for type of drink also differed between genders: men mostly preferred drinking spirits on special occasions and many drank beer as well on ordinary days, and among women wine and sparkling wine were more popular. Few people reported also drinking home produced alcohol such as ‘samogon’ (spirit, usually 40% of ethanol), berry/fruit wine and ‘nastoyka’ (herb or berry-based tonic, usually over 25% of ethanol).

### Traditional drinking pattern

Both men and women reported that they drank on holidays and special occasions, many of which were ritualized; the so-called traditional drinking pattern. Those included major public holidays and celebrations such as New Year, International Women's Day, Victory Day, Day of the Defender, birthdays; more recently, religious holidays such as Easter and Christmas (not widely celebrated during the Soviet regime); weddings and funerals, commemorations of the dead and during visits of friends or relatives as part of the hospitality. In fact, any special occasion was often accompanied with alcohol, which according to both male and female respondents helped conversation and socializing, enhanced mood and helped people to feel joyful. Even abstainers reported keeping alcohol at home for celebrations in case someone visited them. One would be considered ‘a bad host’ if there were no alcohol offered to the guest.

As described in the majority of cases, drinking during special occasions happened around the table at home, always with a lot of food and often with speeches (‘tosty’)—at least for the first two or three drinks.

[We drink] on different holidays. Birthdays [for example]. Guests come, sit down at a table. We have 10 people usually. Then congratulations [follow], speeches. When a speech is told you have to drink. Some drink a shot to the end, some take just a sip, just have a taste. Men usually drink it all at once. Shots … maybe about 70 grams. Then cold appetisers, salads, then main course, pelmeni in general or golubtsy. Then a cake, chocolates, fruit—all table is full. (man, 65)

Women's drinking during special traditional occasions was reported to be different from men's drinking both by female respondents and by male respondents. Women preferred lighter drinks such as wine, sparkling wine, martini and drank in much smaller quantities. It was often cited that women ‘only take a sip from a small shot’ (‘prigubit iz rumochki’) or drink one glass of wine/sparkling wine during the whole evening.

I never liked vodka. I do not like its taste. If it is someone's birthday [and there is no wine served], it is customary to celebrate it, I could drink at most 25 grams [of vodka] and then eat well. On the most holidays I prefer wine or sparkling wine. (woman, 54)

For women we usually take a bottle of martini. That is for five of them. And they do not even finish it. Usually something left … Women in general in my surroundings drink wine or sparkling wine and very little. (man, 65)

Men, on the contrary, mostly preferred vodka and drank larger quantities. The first two or three shots are usually drunk by everyone but, the further pace of consumption was reported to be established by everyone individually. The dosage reported by the male respondents during one special occasion averaged at 250 g of spirits. In fact, the majority of men perceived this quantity as a moderate amount when drunk during the whole evening/holiday celebration:

When it is a celebration, a table is full of food, good company; why not to drink a bottle [of vodka]. But of course during 3, 4, 5 h. (man, 50)

Another traditional ritualized drinking reported mostly by men was drinking after banja (steamed sauna). Some people had their own banjas (in private houses) and used it with family and friends, and other men were visiting banjas in the city exclusively in male company. The drinks and amounts varied, although the majority of men reported drinking beer accompanied with dried fish (vobla), women drank beer and wine. Exclusively men reported traditional drinking during such occasions as fishing, hunting and during or before sport events such as hockey or football. These occasions themselves were perceived to be predominately male.

### Individual drinking patterns

Although the majority of the responders reported traditional drinking pattern on special occasions, almost every man but only few women reported different individual patterns as well. In fact, in the beginning of conversation only traditional patterns were reported (e.g. ‘I drink only on holidays’) but as discussion proceeded, more patterns were revealed, especially among men who often did not count drinking ‘small’ amounts (e.g. 500–1000 ml of beer), which they could drink during the week after work or on weekends. Female respondents, on the other hand, did not count ‘a glass or two of beer on a hot day’. For the majority of female and male respondents, these individual drinking patterns were used as means to relax both physically and mentally, to relieve stress and tiredness after work, ‘to calm down the nerves’, to rest.

Individual drinking patterns reported among men included: (a) drinking after working week (on Friday, or other day after the working shift) with colleagues or alone at home to relax, to relieve stress; often larger amounts 250 ml of spirits or 1.5–2.5 l of beer; (b) drinking 50–100 ml of spirits or glass of wine, bottle of beer, after work with dinner every day to overcome tiredness or for better digestion; (c) drinking on weekends not related to the working week, e.g. among pensioners, to mark the end of the week and the beginning of the weekend, mainly beer; (d) drinking once a month when the pension or salary is received; (e) drinking about once a month or less with friends to socialize; (f) drinking during weekends at dachas (summer houses) during summer seasons usually beer (2–2.5 l per occasion); and (g) drinking while at work. In some cases several patterns overlapped.

I drink usually when I receive the pension—it is a small holiday. We buy food and I drink chekushku [a quarter of a litre of vodka which one can buy in a glass bottle], 250 g. Beer I drink about once a week. It is sold in big plastic bottles, 1.5 l each. So, I usually finish it during the evening watching TV (man, 69)

Only a few female responders reported individual patterns apart from drinking on special occasions. Apart from two respondents, the women reported drinking small amounts of alcohol and usually not on a regular basis. The patterns included: (a) drinking a glass or two of beer when it is hot during summer season at the dacha (summer house); (b) drinking a glass or two of wine with female friends once or less than once a month to socialize; (c) drinking from time to time during dinner with the husband (100–150 ml of spirits); (d) drinking after a working week alone at home to relieve stress (250 ml of spirits); and (e) drinking beer on weekends at home alone to relax (1.5 l).

Besides holidays we drink sometimes during Saturday with lunch, or on any other day when we want to relax, always with dinner. And beer of course during summer, when it is very hot and you want to ease the thirst (woman, 54)

We gather together (I have three [female] friends) about once a month to share news, some events, to relax. Three of us finish one bottle of wine during the evening. It became a ritual for us. You know, every one is busy, everyone has a job, a family, problems—all these are very tiring … So, when we meet, we know that we will have two-three hours for ourselves—it is a very pleasant feeling (woman, 54)

Table 3 summarizes reported drinking patterns in men and women.

**Table 3.** Common patterns of drinking in different occasion, by gender, as identified by qualitative interviews (+/− denotes presence/absence)

	Men	Women
Traditional drinking patterns		
* *State holidays, family special events	At least three shots of 50 ml of spirits (average 250 ml)	A sip, one glass of wine/sparkling wine
Banja (bath)	+ beer/vodka	+ beer/wine
Hunting	+ vodka	−
Fishing	+ vodka	−
Life sport events (usually football or hockey)	Beer, fortified wine, brandy and vodka	−
Individual drinking patterns		
Drinking after working week/shift	+ vodka, beer; 250 ml of vodka or 1.5–2.5 l of beer	+ vodka, beer
Drinking on weekends not related to work	+	−
Drinking once a month when pension or salary received	+	−
Drinking small amount after work with dinner every day	+50 ml of vodka, or glass of wine, bottle of beer	−
Drinking while at work	+	−
Drinking during weekends at dachas (summer houses) during summer	+2–2.5 l of beer	+ one to two glasses of beer
Infrequent (once a month or less) drinking with friends	+250–500 ml of vodka	+ two to three glasses of wine
Infrequent drinking during dinner with a spouse	−	+100–150 ml of vodka

The difference in drinking patterns was also shown in reports about how the significant other drinks. About half of women respondents reported that their husbands had problems with alcohol such as ‘zapojs’ (drinking non-stop for more than 2 days), having hangovers and drinking in the morning, and receiving treatment for alcohol dependence. In fact, some women reported that very heavy drinking by their significant others influenced their own alcohol intake in a way that they ‘could not stand alcohol’ at all, and never kept it at home. Only one man reported that his former wife ‘drank a lot’.

### Perceived reasons behind the gender differences in drinking

The main reason given why women drink much less than men was a traditional role of woman as a mother and a keeper/carer of house and family, which greatly increase women's responsibilities after work in comparison with men. As a result, women become very busy, have almost no spare time and have fewer occasions to drink. It was also reported that the woman has to provide an example of order by her own behaviour, and sometimes needed ‘to control’ her husband's drinking. These expectations would decrease women's drinking even during traditional drinking occasions.

You are the woman and you have to keep your female image, you have to show an example for your children, for your husband, and for others. You have to be clean, you have to do laundry, to cook and to clean, you have to find time to do everything. It does not mean that you should not drink at all but [when there is a drinking occasion] you just sip a bit [from a glass] and put it back. You have to be ideal. (woman, 68)

If you are a woman you should be a woman. You should not drink every day or without any particular reason or occasion. You have a family, your household responsibilities (man, 65)

The other reason given (mainly by male respondents) was that women have less physical ability to drink. Women get drunk faster than men and if they drink on the same level as men they become drunkards fast. At the same time, the image of a drunken woman was perceived with much more negative attitude than the image of a drunken man by both male and female respondents. To be drunk was considered to be ‘not feminine’ and did not correspond to the image of women, as ‘a woman should be a woman’. It was reported that female alcoholics look ‘much uglier’, ‘disgusting’, ‘abnormal’, and for them it is much more difficult to stop drinking even with medical help.

If for men maybe it is appropriate to stumble drunkenly, that is understandable. But when you see a [drunk] woman it is very unpleasant. No questions about it. If people see a drunken man they can smile. But when a woman is drunk it is a terrible scene. People always judge it. (woman, 48)

The reasons behind men's drinking were opposite to those of women. It was reported that men can drink more first of all because they are ‘stronger’ in physical terms, and second of all they do not have as many domestic responsibilities as women. Men's social role as a main breadwinner for the family kept them ‘immune’ from responsibilities at home, limiting their tasks to ‘fixing things when they are broken’. Hence, men have more time and have more occasions to drink.

[Men] have almost no responsibilities at home. It is good if he has a summer house and he is busy fixing it, or a car and garage. But if there are no such things there is practically nothing for them to do. And it spoils them to such a degree that they do not want to even get up from the sofa … And of course they have more free time and can afford to get drunk. (woman, 49)

Finally, it was mentioned by the majority of participants that current trends between genders are changing, and the younger generation drinks in a different way. It became customary for both young men and young women (‘molodegh’) to drink beer almost every day and at the same level and often on the street and other public places. This was linked by participants with changed values, increased advertising and access to alcoholic beverages:

The moral criteria became different. Earlier it was a very rare occasion to see a young girl drinking beer at a bus stop, and people would treat her with a certain [negative] attitude. But now all you see is beer, beer, beer. I think it is not attractive. And does she not have a home where she can drink this can of beer? (man, 49)

## DISCUSSION

It is well documented that cultural norms and customs regulate consumption of alcohol throughout the world. Who, when, where, how and what to drink is regulated in many societies (Heath, 1998; Room, 1997). In the case of gender, drinking often becomes a symbol of gender roles, and societies identify and express gender roles through drinking (Holmila *et al*., 1990; Holmila and Raitasalo, 2005; Room, 1996; Wilsnack and Wilsnack, 1997). Women traditionally are expected to drink less than men. Women are condemned when they drink and could lose their traditional roles as caretaker and moral agent (McDonald, 1994). Women's family responsibilities, with their maternal and nursing roles, are not compatible with drinking, and so women have more to lose than to gain from it (Room, 1989).

Our study confirmed previous research findings which have shown large differences in drinking between men and women in Russia. Women had fewer drinking occasions per year, they had lower amounts of alcohol consumed both annually and weekly, had significantly fewer problems related to alcohol and had significantly fewer occasions of binge drinking. The differences between men and women were not explained, even partly, by controlling for socio-demographic variables.

Qualitative data unveiled what lay behind patterns identified in the epidemiological study in detail. It showed that women were expected to drink and drank much less during mostly cited traditional drinking occasions. Moreover, when individual patterns were described these differences persisted: men had more occasions to drink, drank in larger quantities and consumed stronger alcohol such as vodka, whereas women had many fewer opportunities to drink and when drinking chose ‘lighter’ alcohol and smaller quantities. It is worthwhile to note for future research studies that individual patterns were often not reported in the beginning of the conversation but only after  prompting, and perceived small amounts of alcohol, especially beer (e.g. a 500 ml can of beer), were not counted as a drinking event.

The expectations of drinking behaviour also were reflected in attitudes towards drunkenness for men and women. If the appearance of a drunken man in public was tolerated, drunken women were always negatively judged. ‘To drink alcohol’ was simply perceived as not feminine as ‘a woman is supposed to be a woman’. On the other hand, for male respondents, drinking often large quantities of alcohol was perceived as quite normal. It seemed that this particular division of the behaviour came of the fact that ‘things are the way they are by virtue of the fact that men are men and women are women’ (West and Zimmerman, 1987), confirming the notion of gender display through drinking (McCreary *et al*., 1999; Room, 1997; Wilsnack *et al*., 2005), and showing that alcohol consumption is highly conventionalized between genders in this sample, which is consistent with the findings of both quantitative and qualitative studies conducted in Russia (Abbott *et al*., 2006; Cockerham *et al*., 2006; Van Gundy *et al*., 2005).

Our qualitative findings also have shown that besides a reported physical ability for men to drink more, gender roles and strong traditional culture around women's drinking affected drinking behaviour in this sample. Women's domestic responsibilities left them much less spare time, and their role as a caretaker and a ‘controller’ of husband's drinking implied sobriety. Men's main role as ‘bread winner’ and traditional limited involvement in domestic responsibilities created more occasions to drink during leisure and as a means to relieve stress and tiredness after work.

The Soviet pursuit of equality for both genders increased women's participation in the work force dramatically in Russia, but this has not relieved them from their traditional role of family caretaker, and ‘the strong male-breadwinner family model’ is still supported by the majority of the Russian population (Kiblitskaya, 2000; Motiejunaite and Kravchenko, 2008; Shiraev, 1999; Van Gundy *et al*., 2005). In fact, it has been shown that a traditional, patriarchal model, often called ‘re-masculinization’, has been promoted since the fall of the Soviet Union (Ashwin, 2000; Shiraev, 1999; Watson, 1995). This persisting traditional gender structure has left women with a ‘double burden’ of working and family caring and men have remained distant from domestic tasks (Cubbins and Vannoy, 2005; Goodwin and Emelanova, 1995; Sandnes, 2008). It has been reported in Russia and in some other eastern European countries that the double burden for women does decrease women's drinking and ‘protect’ women from heavy drinking (Ahlstrom *et al*., 2001; Ashwin, 2007). On the other hand, males’ sole role of a breadwinner puts them at greater risk of heavy drinking, especially in times when this role cannot be adequately fulfilled (Ashwin and Lytkina, 2004; Kiblitskaya, 2000; Watson, 1995). It appears, therefore, that to combat cultural permissiveness or encouragement of male drinking, it will be necessary either for society to undergo a radical shift across a wide range of mores (which seems unlikely in the near future) or that public health campaigns will need to focus on dissociating alcohol from these cultural expectations and values. This will require an extended and concerted effort and considerable resources, but given the damage that male alcohol consumption is doing to the Russian economy as well as to the health and wellbeing of its population, such expenditure would probably be more than repaid.

### Limitations

Several limitations of our study should be considered when interpreting the results. First, the present study investigated a relatively restricted age range, focusing on middle-aged and older participants. In most populations, drinking (and particularly problem drinking) tends to decrease with age. Thus, the differences in drinking found in this study may not apply to younger persons. The emergence of beer and light alcoholic beverages markets and consumer hedonistic culture among young people are likely to decrease the gender gap in drinking in Russia, an area that is yet to be studied. Moreover, most of the participants were brought up and spent most of their adult life during the Soviet era, which is culturally quite different from post-Soviet times; that might correspond to different gender expressions and gender identities and related drinking behaviour. For example, one study has shown that young women in post-Soviet Russia perceive the social environment as more permissive of alcohol consumption than during the Soviet era, and could be more involved in drinking behaviour to exercise their personal freedoms in new modern times (Hinote *et al*., 2009).

Second, drinking is typically under-reported in surveys and that is likely to be the case here. The absolute levels of means and proportions estimated in our study may, therefore, be underestimated. In addition, there is some evidence that in Russia, women tend to under-report their alcohol intake to a larger extent then men (Laatikainen *et al*., 2002)—this may lead to overestimation of the gender gap in drinking. However, the differences between men and women were so large that it is extremely unlikely that they could be explained by differential under-reporting. The validity of the quantitative findings view is supported by analyses of serum gamma-glutamyl transferase, which show a gender difference consistent with self-reported alcohol intake (Nikitin *et al*., 2008).

Third, the qualitative study can only access the perceptions of participants, and it may be that there are important influences operating that are outside their awareness. For example, it has been shown in a number of populations that price plays a major role in consumption (Wagenaar *et al*., 2009), but this issue was not included in the semi-structured interview and, interestingly, it was not mentioned by the participants.

Finally, the quantitative data were collected in 2002–2005 and patterns may have changed since then. This is unlikely, however, as the qualitative data were collected during re-examination of the cohort, and the self-reported drinking showed a reasonable stability over time.

## CONCLUSIONS

Our study has shown a large gap in drinking in Novosibirsk between genders. It showed that traditional drinking culture is very different between men and women, with women expected to drink much less or only ‘symbolically’. Drinking large amounts and being drunk were more tolerated amongst men. Gender roles were the main reason behind drinking practices and in some cases, very heavy drinking by men was balanced by near abstinence among women as someone had to take care of the family. Moreover, our study detected a variety of individual patterns which, to our knowledge, have not previously been adequately documented. These patterns also might be less likely to be reported as most reported drinking is traditional ‘on special occasions [po prazdnikam]’. This is especially true of drinking perceived small quantities of beer, which participants did not count as ‘a real drink’.

## Funding

The study was supported by the Wellcome Trust [064947/Z/01/Z and 081081/Z/06/Z].
